# Increased Chance of Live Birth Following Use of Connected Ovulation Test System: Outcome Results from a Randomized Controlled Trial

**DOI:** 10.1089/whr.2021.0102

**Published:** 2022-01-31

**Authors:** Sarah Johnson, Sharon Bond, Bola Grace, Lorrae Marriott

**Affiliations:** SPD Clinical Research Department, SPD Development Company Limited, Bedford, United Kingdom.

**Keywords:** Clearblue Connected Ovulation Test System, conception rate, live birth rate, luteinizing hormone, ovulation testing, pregnancy

## Abstract

***Background:*** Natural conception requires intercourse to occur during the fertile window of a woman's menstrual cycle. This follow-up study of a randomized controlled trial aimed to determine whether the use of a urine ovulation test system, which tracks elevations in both luteinizing hormone and an estradiol metabolite, increases the likelihood of live births in women trying to conceive.

***Materials and Methods:*** In the home-based trial, 844 women aged 18–40 years who were attempting to conceive were randomized 1:1 into the test or control arms. Volunteers participated for up to two full cycles and conducted digital pregnancy tests, collected urine samples, and kept a menstrual diary to determine pregnancy status. In this follow-up, all pregnant volunteers were asked to complete a form on final pregnancy outcome.

***Results:*** Overall, 247 (29.3%) of the 844 volunteers reported a pregnancy; final outcome data were available for 198 pregnancies. For cycle one, the live birth rate was 16.4% for the test group and 8.5% for the control group (odds ratio: 2.12; 95% confidence interval [CI]: 1.34–3.35; *p* = 0.001). For cycles one and two combined, the live birth rate was 24.5% and 17.5% for the test and control groups, respectively (odds ratio: 1.53; 95% CI: 1.07–2.19; *p* = 0.023). The proportion of miscarriages was not significantly different between both groups and 78% of pregnancies resulted in a live birth.

***Conclusions:*** The increased conception rate observed following the use of the Clearblue Connected Ovulation Test System was found to translate into an increased live birth rate.

Clinical Trial Registration number: NCT03424590.

## Introduction

A woman's desire to have a baby may be related to various factors, such as her values, goals, employment status, and financial and emotional circumstances.^1^ These factors may account for the increasing number of women in developed countries who are delaying attempts to have a baby until they reach a period in life when raising children is consistent with both their career and life aspirations.^2,3^ This delay has meant that many women are now attempting to conceive when their fertility is already in decline, which occurs at a more rapid pace once a woman reaches her mid-30s.^4,5^

Natural conception requires intercourse to occur during the fertile window of a woman's menstrual cycle.^6,7^ The fertile window comprises the 5 days preceding ovulation as well as the day of ovulation itself.^6,7^ In addition to influencing chances of conception, it has been suggested that the timing of intercourse has an impact on miscarriage rates as studies have shown that conceptions that are distant (±3 or more days) from the estimated day of ovulation carry a higher risk of miscarriage.^8–10^

Miscarriage, generally defined as pregnancy loss before 20 weeks of gestation, affects up to 25% of all pregnancies, with more than 80% of miscarriages occurring within the first 12 weeks of pregnancy.^11–13^ In order for women to be effective at timing intercourse in relationship to the fertile period, a good awareness of their ovulation day is beneficial.^14^ Findings from a study on women of reproductive age reported that fertility knowledge, specifically relating to topics such as ovulation, conception, and miscarriage, is limited.^15^

Fertility-tracking applications (apps) typically associated with smartphones can be used to track the menstrual cycle.^16^ As these apps can predict the ovulation day, they are often aimed at and marketed to women who wish to either achieve or avoid pregnancy.^16^ However, there are variations of menstrual cycle characteristics, including the day of ovulation, that exist even in women with regular cycles.^17^ Because of the variability of menstrual cycle length, the reliability of calendar-based fertility apps has been questioned.^17,18^

Timing intercourse to occur during the fertile window can be facilitated by monitoring key fertility hormones, such as the luteinizing hormone (LH) and the estradiol metabolite, estrone-3-glucuronide (E3G).^19,20^ Changes in hormone levels enable the approximation of the time of ovulation; LH levels surge the day before ovulation and urinary E3G levels rise in the 5 days preceding ovulation.^21^ E3G has been demonstrated to be a useful urinary marker for predicting the fertile window, while LH is the best predictor of imminent ovulation, and both hormones can be monitored using home-based ovulation tests.^20,22,23^

The Clearblue Connected Ovulation Test System (Swiss Precision Diagnostics [SPD] GmbH, Geneva, Switzerland) is designed to be used by women at home, and is able to accurately predict the fertile window by tracking elevations in both LH and E3G that precede ovulation.^20^ The test system can be connected via Bluetooth to the user's smartphone or tablet, where the app records information relating to the woman's menstrual cycle and uses this information to determine when urine tests should be conducted.^20^ Through urine hormone measurements, the test reports one of three levels of fertility: low fertility when hormone levels are at baseline; high fertility when the monitor detects increasing E3G levels; and peak fertility upon detection of the LH surge.^20^

The use of ovulation tests to improve the timing of intercourse has been shown to increase the chance of conception.^22^ In the previously reported randomized controlled trial of 844 women aged 18–40 years attempting to conceive, it was found that using a urine ovulation test system to time intercourse increased the likelihood of conceiving within two menstrual cycles, with higher conception rates in the test group, and with cycle one having an odds ratio of 2.0 and cycles one and two (combined) reporting an odds ratio of 1.4.^20^

However, the impact of ovulation test use on pregnancy viability and miscarriage is unknown, and the trial did not report on how many of the pregnancies resulted in live births. These data are important because, for those attempting to conceive, the ultimate goal is not pregnancy, but rather the birth of a healthy baby. This follow-up study investigated the pregnancy outcomes of the original study population and determined whether the increased likelihood of conception using the Clearblue Connected Ovulation Test System is carried through to an increased likelihood of a live birth among women attempting to conceive, compared with those not using the test system.

## Materials and Methods

### Study design, inclusion and exclusion criteria, and volunteers

This study was a follow-up to an open-label, home-based, randomized controlled trial (clinical trial number: NCT03424590) of women aged 18–40 years who were actively attempting to conceive.^20^ A total of 1000 women were recruited from England, Wales, and Scotland to conduct the study in their own home, of whom 436 were randomized into each arm of the first cycle of the study. The study was approved by the SPD Ethics Committee on January 17th, 2018 (protocol 0987) and all procedures were conducted in accordance with relevant regulations and guidelines.^20^

Full methodology regarding the study inclusion and exclusion criteria and the pregnancy rate endpoints of this randomized controlled study have previously been published.^20^ As previously reported, volunteers were randomized 1:1 into the test or control arm, stratified by the age of the volunteers, with two cohorts (<35 and ≥35 years of age). Volunteers assigned to the test group were required to use the test system in their homes, according to instructions provided, for up to two complete menstrual cycles. Women assigned to the control group were required to continue attempts to conceive but were told not to use any urine ovulation tests for the duration of the study.^20^

It has previously been reported that volunteers were provided with digital urine pregnancy tests, urine sample pots, and a form to record menses to determine pregnancy status at the end of each cycle. Volunteers were asked to collect a urine sample and conduct a pregnancy test on specified test days to determine pregnancy status. Those with amenorrhea were classed as “not pregnant” in the efficacy endpoint analyses.^20^

For this follow-up study, all pregnant volunteers were emailed a pregnancy outcome form after their predicted due date to provide details on the outcome of the pregnancy. The questionnaire asked if the pregnancy resulted in a live birth, and if so, data on the date and mode of delivery were collected, along with the sex and birth weight of the baby/babies. For those women whose pregnancy did not result in a live birth, they were asked to indicate the reason for the end of their pregnancy from the following list: still birth, miscarriage, ectopic pregnancy, elective termination, or other.

### Follow-up trial endpoints

This follow-up study aimed to determine, across one and two cycles, the difference in live birth rates between volunteers attempting to conceive in the home setting using the urine ovulation test system and those not using a urine ovulation test.

### Statistical analysis

**FIG. 1.**
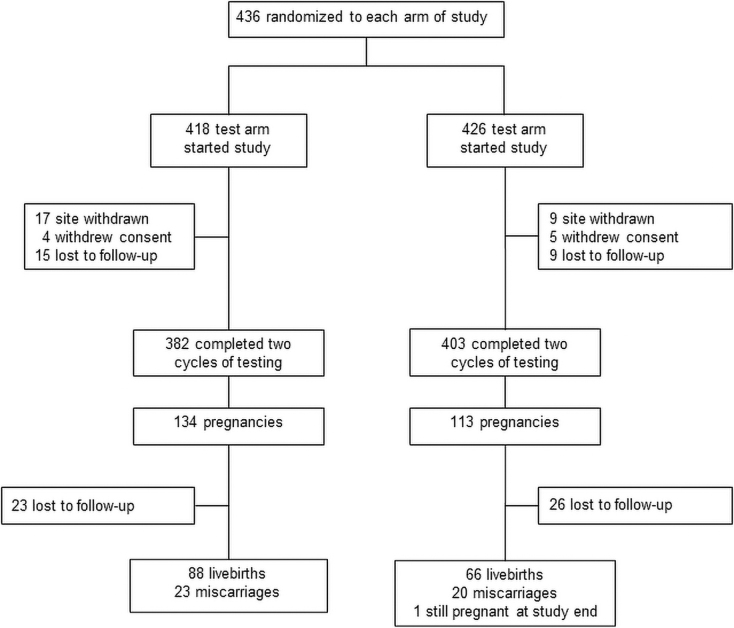
Flow diagram of the number of volunteers participating in the study.

Over 400 volunteers per arm were randomized to meet the required sample size of 346 volunteers per arm, with an expected dropout rate of 10%. This sample size was calculated assuming a pregnancy rate of 25% in the test group, with an odds ratio of 1.9, to give a power of 90% with a significance level of 5%. Participant distribution for the study is shown in Figure 1.

The endpoints for each cycle were calculated using a Fisher's exact test 2 × 2 contingency table. The proportion of live births and pregnancies that did not result in live births in each arm after two cycles was calculated for the population of volunteers not lost to follow-up. The odds ratio was calculated based on the formula: [LBt/(1–LBt)]/[LBc/(1–LBc)] where the proportion of volunteers whose pregnancies resulted in live births in the control group is denoted as “LBc” and the proportion of volunteers whose pregnancies resulted in live births in the test group is denoted as “LBt.” Two sample *t*-tests were used to determine the *p*-value for the birth weight and gestational age of the infants in the test and control groups, while a Fisher's exact test was used to determine the sex *p*-value.

## Results

Overall, 418 of the 436 women randomized to the test arm and 426 of the 436 women randomized to the control arm began the study (Fig. 1). A total of 247 pregnancies were reported from the volunteers over the two cycles.^20^ Table 1 displays the demographics of the pregnant volunteers who completed the study and reported a pregnancy outcome in the test arm (*n* = 134) and the control arm (*n* = 113).

**Table 1.** Demographics of the Pregnant Volunteers Who Completed the Study and Reported a Pregnancy Outcome in the Test and Control Arms

Demographics	Test arm *N* = 134	Control arm *N* = 113
Age, years, median (range)	30 (21–40)	30 (19–40)
Self-reported cycle length, days, median (range)	29 (20–38)	29 (21–40)
Shortest self-reported cycle length, days, median (range)	27 (19–33)	28 (18–40)
Longest self-reported cycle length, days, median (range)	31 (26–40)	31 (27–42)
Weight, kg (mean ± SD)	72.94 ± 16.71	69.44 ± 14.76
Height, m (mean ± SD)	1.65 ± 0.06	1.65 ± 0.06
Body mass index, kg/m^2^, (mean ± SD)	26.67 ± 5.62	25.43 ± 5.00
Smoking status, *n* (%)		
Current	2 (2.3)	2 (3.0)
Previous	20 (22.7)	11 (16.7)
Never	66 (75.0)	53 (80.3)
Taking folic acid, *n* (%)		
Yes	75 (85.2)	55 (83.3)
No	13 (14.8)	11 (16.7)
Don't know	0 (0.0)	0 (0.0)
Used contraception in the last 12 months, *n* (%)		
Yes	69 (78.4)	47 (71.2)
No	19 (21.6)	19 (28.8)
Months since stopping contraception (median, range)	3 (1–10)	4 (1–11)

SD, standard deviation.

### Proportion of live births among test versus control groups

Of the 844 volunteers, 247 (29.3%) reported achieving pregnancies. Final outcome data were available for 198 of the 247 pregnancies (lost to follow-up: 17.2% from test group and 23.0% from control group). After one cycle, 59 live births were recorded in the test group analysis set of 359 volunteers, giving a live birth rate of 16.4%. This was significantly higher than the 32 live births out of 377 volunteers, for a live birth rate of 8.5% in the control group, giving an odds ratio of 2.12 (95% confidence interval [CI]: 1.34–3.35; *p* = 0.001 by Fisher's exact test; Table 2).

**Table 2.** Live Births Recorded for Test Versus Control Groups in the Analysis Set of the Study Population, Excluding Those Lost to Follow-Up

	Test group *N* = 359 (*n*, %)	Control group *N* = 377 (*n*, %)	Odds ratio (95% CI)	Fisher's exact test *p*-value
One cycle: Total live births	59 (16.4) (95% CI: 12.8–20.7)	32 (8.5) (95% CI: 5.9–11.8)	2.12 (1.34–3.35)	0.001
Two cycles (combined): Total live births	88 (24.5) (95% CI: 20.1–29.3)	66 (17.5) (95% CI: 13.8–21.7)	1.53 (1.07–2.19)	0.023

CI, confidence interval.

No significant difference in live birth rates was seen between the test and control groups in the second cycle. Across the two cycles cumulatively, the live birth rate for the test group was 24.5% compared with 17.5% for the control group, giving an odds ratio of 1.53 (95% CI: 1.07–2.19; *p* = 0.023 by Fisher's exact test; Table 2).

### Proportion of miscarriages among test versus control groups

The proportion of miscarriages was not significantly different between the test and control groups. In the test group, out of the 111 pregnancies for which outcome data were available, 23 miscarriages were reported (20.7%), whereas for the 87 pregnancies for which outcome data were available for the control group, 20 miscarriages were reported (23%) (*p* = 0.731 by Fisher's exact test). Overall, 78% of pregnancies resulted in a live birth.

### Other outcome data among test versus control group

In the test arm of the study, there was only one set of twins delivered. The birth weight of the babies delivered in both the test and control groups did not significantly differ, nor was there a difference in the proportion of each sex (Table 3). A difference in gestational age at delivery, when assigned by using the last menstrual period, was seen between the cohorts; those in the test group had delivered on average 5 days earlier than those in the control group (274.8 ± 15.11 days vs. 280.0 ± 15.40 days [mean ± standard deviation]; *p* = 0.038 by Fisher's exact test). There were more cesarean deliveries in the test group (31.7%) compared with the control group (22.8%), while pregnancy induction was more likely to occur in the control group (34.9%) compared with the test group (22.7%).

**Table 3.** Comparison of Live Birth Outcome Data for Test Versus Control Groups

	Test group *N* = 88^a^	Control group *N* = 66	*p*
Birth weight (g)			
Mean ± SD	3433.5 ± 562.2	3385.8 ± 528.2	0.652^b^
Range	1620‒4930	2100‒4650	
Sex, male, *n* (%)	47 (53.4)	34 (51.5)	0.871^c^
Gestational age (days since LMP)			
Mean ± SD	274.8 ± 15.1	280.0 ± 15.4	0.038 ^b^
Range	210‒308	237‒354	
Delivery type, *n* (%)			
Vaginal	40 (45.5)	28 (42.4)	0.745^c^
Elective cesarean	15 (17.0)	10 (15.2)	0.827^c^
Emergency cesarean after labor	5 (5.7)	1 (1.5)	0.239^c^
Emergency cesarean before labor	8 (9.0)	4 (6.1)	0.557^c^
Labor induced, delivery type unknown	20 (22.7)	23 (34.9)	0.106^c^

^a^ For test group, there were 88 live births, which included one set of twins.

^b^ By two-sample *t*-test.

^c^ By Fisher's exact test.

LMP, last menstrual period.

## Discussion

The results from this follow-up study showed that use of the Clearblue Connected Ovulation Test System, which was associated with an increased conception rate in a previous study,^20^ translates into an increase in the rate of live births among women attempting to conceive. After one cycle, the proportion of women who reported a live birth was double that of the control group. After two cycles, a greater proportion of women using the urine ovulation test system had a live birth compared with those in the control group. The odds ratios for a live birth were comparable to the odds ratios reported in the previous study for achieving conception after one (2.1 [95% CI: 1.3–3.4] vs. 2.0 [95% CI: 1.4–2.8]) and two cycles (1.5 [95% CI: 1.1–2.2] vs. 1.4 [95% CI: 1.01–1.9]).

These findings show that in women who used a urine ovulation test system to detect their fertile window improved conception rates translated to an increased likelihood of having a pregnancy that led to a live birth, particularly in the first cycle of use. No significant benefit of using the urine ovulation test system was shown in the second cycle, which is an unexpected result. Additional analyses may reveal why this was the case, and provide insight into further improving the effectiveness of the system.

The results of the previous conception study supported an earlier trial, which showed that use of a home-based fertility tracking monitor increased the likelihood of achieving conception during the first two cycles of use in women who had been attempting to conceive for up to 2 years.^20,23^

However, pregnancy outcome data are the most important data to report for an interventional conception study as women utilizing ovulation test products do so to facilitate the process of having a baby, not simply to become pregnant. In addition, pregnancy outcome is the standard metric that *in vitro* fertilization clinics are required to report,^24^ thus providing consistency with other parts of the fertility field. Despite this, not many studies have reported the live birth rate, especially studies that include the use of fertility tracking apps.

The rate of live births may be influenced by the timing of intercourse, as it has been suggested that if conception occurs outside the fertile window and the fertilizing sperm or fertilized ovum is significantly aged, it may be more likely to result in miscarriage.^25–28^ In the current study, the majority (88.5%) of participants in the test arm reported that they had focused intercourse to a particular part of their cycles, compared with only half of women in the control arm.^20^ However, while conception rates were higher in the test arm, the proportion of miscarriages was not significantly different between the test and control groups. Thus, the current study provides no support for this hypothesis.

Another popular theory, although not supported by current scientific evidence, is that the timing of intercourse can affect the likelihood of having a male or female baby – Shettles' method.^29–31^

It was proposed that for a male child, intercourse should be timed as near to ovulation as possible because the “lighter” Y chromosome-bearing (male) sperm would arrive at the egg ahead of the “heavier” X chromosome-bearing (female) sperm. As reported in the previous study, women in the test arm timed intercourse to ovulation and the test accurately identifies the ovulation day.^20^ Sex ratio was approximately equal in both the test and control groups, and was consistent with that of the wider population (105.2 males per 100 females of births registered in England and Wales)^32^ and therefore, the outcomes of this study did not support Shettles' method.

An unexpected finding of this study is that women in the test group gave birth at an average gestational age of 275 days, which was 5 days earlier than that of the control group and earlier than the average reported figure of 279 days from another UK prospective observational study.^33^ This may be due to the slightly higher cesarean rate of 32.7% in the test group, compared with 22.8% in the control group. As cesareans are usually carried out at or before 39 weeks of gestation,^34^ a higher cesarean rate might translate to a lower average gestational age.

In 2019, cesarean rates in the UK were recorded to be 30% (14% elective, 16% emergency).^35^ The difference in gestational age was not reflected by a difference in birth weights between the two groups, which were comparable to the UK averages of 3316 g for female and 3436 g for male newborns.^36^ Further studies, with an increased sample size, would be required to determine the validity of this observation.

Randomized controlled trials provide robust evidence of the efficacy of an intervention, providing no bias has been introduced and that care has been taken to ensure that behavioral bias was reduced, as reported previously.^20^

The limitations of this study are that use of the connected ovulation test system was only investigated across two menstrual cycles, and therefore does not provide evidence of cumulative pregnancies that result in live births over a longer period of use. There were a number of volunteers lost to follow-up across both the test and control groups, which may have impacted the data reported in the study, but loss between groups was relatively equal, and therefore unlikely to introduce substantive bias to the findings. The study was not powered for the secondary endpoint of pregnancy outcome, and therefore the reduced sample size increases the chance of falsely accepting the null hypothesis of no difference (type 1 error).

For women in the early stages of attempting to conceive, information on the appropriate timing of intercourse is a form of simple and effective advice that can be easily provided.^20^ The information collected by the Clearblue Connected Ovulation Test System can be used to assist with patient management; for example, objective evidence of failure to conceive following 6 months of intercourse timed at ovulation as predicted by the LH surge may suggest the need for an investigation for male factor issues.^20^

## Conclusion

This study found that the higher rates of conception across one and two menstrual cycles associated with the Clearblue Connected Ovulation Test System translated into higher rates of live births when compared with those not using a urine ovulation test system. Additional research should be conducted to identify the determinants that can predict or maximize the chances of not only achieving conception, but also having a live birth among women attempting to conceive.

## Abbreviations Used

CI: confidence interval
E3G: estrone-3-glucuronide
LH: luteinizing hormone
LMP: last menstrual period
SPD: Swiss Precision Diagnostics
SD: standard deviation
